# Development and Preliminary Technical Validation of an Interactive, Surgeon-Oriented Framework for Digital Surgical Occlusion Planning in Orthognathic Surgery

**DOI:** 10.3390/jcm15166322

**Published:** 2026-08-15

**Authors:** Ylenia Gugliotta, Elena Carlotta Olivetti, Giorgia Bruno, Gabriele Maria Galasso, Fabio Roccia, Fabrizio Ferretti, Sandro Moos, Enrico Vezzetti, Federica Marcolin, Guglielmo Ramieri

**Affiliations:** 1 Department of Surgical Sciences, Division of Maxillo-Facial Surgery, AOU Città della Salute e della Scienza, University of Turin, Corso Bramante 88, 10126 Torino, Italy; ylenia.gugliotta@unito.it (Y.G.); fabrizio.ferretti@unito.it (F.F.); guglielmo.ramieri@unito.it (G.R.); 2 Department of Mechanical and Aerospace Engineering (DIMEAS), Polytechnic University of Turin, 10129 Torino, Italy; 3 Department of Management and Production Engineering (DIGEP), Polytechnic University of Turin, 10129 Torino, Italy; elena.olivetti@polito.it (E.C.O.); s319812@studenti.polito.it (G.B.); s319811@studenti.polito.it (G.M.G.); sandro.moos@polito.it (S.M.); enrico.vezzetti@polito.it (E.V.); federica.marcolin@polito.it (F.M.)

**Keywords:** orthognathic surgery, surgical occlusion, virtual surgical planning, virtual occlusion

## Abstract

**Objectives**: To develop and validate a fully digital, surgeon-oriented interactive framework for final dental occlusion alignment in orthognathic patients. **Methods**: A digital framework integrating automatic alignment, using a two-dimensional Iterative Closest Point (2D ICP) algorithm with geometric corrections and optimization-based refinement, and an interactive graphical user interface (GUI) for standardized manual refinement were implemented in MATLAB. Validation was performed on preoperative digital models from 21 orthognathic patients. Obtained digital occlusions were compared with manually articulated physical models, considered the reference standard. Translational and rotational discrepancies were assessed before and after manual refinement. **Results**: Twenty-one patients were included. Manual refinement reduced the greatest translational directional bias along the Y-axis (MV: −2.26 ± 2.1 to −1.17 ± 0.92 mm) and significantly decreased the magnitude of corresponding error (MAV: 2.54 ± 1.73 to 1.26 ± 0.78 mm; *p* = 0.002). Error magnitude also decreased along the X-axis (1.12 ± 0.84 to 0.38 ± 0.31 mm; *p* = 0.002), whereas a small increase was observed along the Z-axis (0.93 to 1.21 mm; *p* = 0.02). Overall translational RMSE improved significantly (1.83 ± 0.91 to 1.09 ± 0.39 mm; *p* = 0.001). Among rotational components, yaw showed the greatest reduction in error magnitude (MAV: 2.61 ± 1.91° to 0.83 ± 0.71°; *p* = 0.001), while no statistically significant changes were detected for pitch (*p* = 0.09) or roll (*p* = 0.13). Rotational RMSE decreased from 2.00 ± 1.01° to 1.35 ± 0.25° (*p* = 0.01). **Conclusions**: The proposed pipeline was completed for all cases. Automatic alignment provided a reliable starting point, but standardized manual refinement remained essential.

## 1. Introduction

The assessment of the final dental occlusion represents a critical step in orthognathic surgery planning [1,2,3,4,5,6], as achieving a stable and functional occlusal relationship is essential to ensure dental stability, periodontal health, and temporomandibular joint function, as well as efficient mastication, phonation, and overall orofacial balance [7,8,9]. Consequently, precise occlusal assessment is fundamental for identifying the source of discrepancies and selecting the most appropriate treatment strategy.

Although manual model articulation remains a well-established clinical approach for defining the target surgical occlusion [10,11,12], previous studies have highlighted several inherent limitations of conventional manual workflows, including patient discomfort during impression taking, the risk of impression deformation, the need for physical space for model fabrication and storage [10,12,13,14], and, importantly, operator dependence with potential inter- and intra-operator variability [5,15].

Therefore, manually established occlusion should be regarded as a clinically relevant reference rather than an absolute, error-free gold standard when validating digital occlusal workflows.

Recent advances have significantly transformed orthognathic surgery workflows, promoting the increasing adoption of digital and computer-assisted planning [13,14,15,16,17,18,19,20].

The integration of digital dental models in .STL format into surgical planning software enables the creation of a fully virtual environment in which osteotomies and skeletal movements can be simulated, alternative treatment strategies compared, and the most appropriate solution for each patient selected [6,10,21,22,23,24].

Despite these advances, conventional workflows often still rely on the fabrication of physical models derived from intraoral scans for occlusal planning [25,26]. In this process, resin models of the maxillary and mandibular arches are produced and manually articulated by the surgeon to reproduce the desired occlusion and are then rescanned to record the resulting relationship [27,28]. These data are subsequently imported into dedicated software for surgical planning. However, this workflow remains time-consuming and resource-intensive due to the need for physical model fabrication.

Although several virtual occlusion (VO) tools have been introduced, their widespread clinical adoption remains limited by the lack of tactile feedback and by the absence of physical collision constraints in the virtual environment, where the operator has to rely exclusively on visual indicators, such as occlusograms and colour-coded contact maps, to evaluate occlusal relationships [6,10,12,29,30,31,32,33].

Several authors have focused on the development and validation of fully automated algorithms for virtual occlusion. Although these approaches have shown promising accuracy when compared with manually established occlusion, their translation into routine clinical practice has remained limited, mainly due to workflow complexity and limited usability in everyday clinical settings [34,35,36].

Alternatively, augmented reality- and mixed reality-based systems have been proposed to support virtual occlusion planning; however, these approaches typically require dedicated hardware and additional equipment, which may limit their accessibility and integration into routine clinical workflows [12,31].

Importantly, the validation of these tools has largely focused on geometric agreement with a reference occlusion rather than on clinical outcomes. Indeed, clinical validation is inherently more complex, as the final surgical result depends not only on the definition of the virtual target occlusion but also on its accurate intraoperative transfer. The accuracy of this transfer may be influenced by several factors, including splint manufacturing and positioning, condylar seating and fixation. Therefore, the geometric accuracy of the virtually defined target occlusion and the accuracy with which this target is transferred to the patient should be considered as distinct components of the overall surgical workflow.

Existing digital solutions include both automated alignment procedures and operator-driven virtual articulation tools, as well as hybrid approaches combining automated alignment with subsequent manual refinement. However, these solutions are generally integrated into dedicated commercial software platforms, potentially limiting their accessibility and requiring additional software acquisition. In this context, the proposed framework was developed as an in-house solution that can be implemented on a standard personal computer equipped with MATLAB, without requiring dedicated commercial software for orthognathic surgery planning.

The present study aims to develop and validate a fully digital surgeon-oriented interactive interface for dental occlusion definition and analysis, designed to be used autonomously by any operator on a standard personal computer. The proposed framework was designed to combine the efficiency of automated alignment with direct surgeon control over the final occlusal relationship, while maintaining the entire procedure within a digital environment and avoiding the need for physical model fabrication and rescanning.

## 2. Materials and Methods

The study was conducted through a collaboration between the Maxillofacial Surgery Unit of AOU Città della Salute e della Scienza, University of Turin, Italy, and the Department of Management and Production Engineering (DIGEP), Polytechnic University of Turin.

### 2.1. Patient Selection

Dental models from patients with occlusal discrepancies and undergoing orthognathic surgical treatment between October 2025 and January 2026 were collected.

The inclusion criteria for the analysis were (1) age ≥ 18 years; (2) presence of an occlusal discrepancy requiring surgical correction; (3) availability of preoperative dental models in .STL format. Exclusion criteria were applied as follows: (1) noisy or incomplete IOS acquisitions; (2) presence of fewer than six pairs of antagonistic teeth per side; (3) segmental surgery of the maxilla; (4) inability to retrieve clinical information.

For each subject, demographic (gender and age) and clinical data (type of facial dysmorphism and surgical procedure) were collected, together with the .STL file representing the dental arches and final MrO, which was considered the reference standard. The dataset comprised patients with a broad spectrum of skeletal and dental malocclusions. No subgroup analysis based on the surgical approach (single jaw versus bimaxillary surgery) was conducted, as the definition of the final occlusion is independent of the number of jaws undergoing surgical repositioning.

### 2.2. Data Acquisition

For each patient, preoperative dental arches and surgical occlusion defined using the manual method on physical plaster casts (Manual Reference Occlusion, MrO), were acquired in digital format using an Aoralscan 3 intraoral scanner (SHINING 3D Tech Co., Ltd., Hangzhou, China). The scanner was calibrated according to the manufacturer’s instructions before each acquisition session, and the articulated casts were scanned following the standard full-arch acquisition protocol. The previously reported full-arch accuracy of the Aoralscan 3 of approximately 170 μm [36] was considered acceptable for the purposes of the present study.

The MrO was established on physical plaster casts by the operating senior surgeon, who was responsible for the surgical planning of each individual case, and it was stabilized using adhesive wax prior to digitization to prevent any displacement during the scanning procedure. As part of the routine preoperative workflow at our institution, the planned surgical occlusion was discussed and confirmed during multidisciplinary preoperative meetings involving the head of the department and junior maxillofacial surgeons. Therefore, the MrO used as the reference standard corresponded to the occlusion that was ultimately adopted during surgery. Subsequently, VO was generated by the same senior surgeon using the proposed interface, with the assistance of a junior surgeon for management of the digital platform. To minimize potential recall bias, digital refinement was carried out at least one month after completion of the conventional manual planning, without access to the reference occlusion. Finally, the resulting VO three-dimensional (3D) meshes were exported in .STL format for further processing.

A digital surgeon-oriented interactive interface was developed incorporating a two-step workflow: (1) a geometry-based pipeline for initial automatic positioning of the dental arches followed by (2) a dedicated Graphic User Interface (GUI) enabling the operator to adjust the occlusion through a surgeon-oriented standardized refinement phase.

### 2.3. Geometry-Based Automatic Pipeline

The first step of the proposed methodology relies on the development of an automatic pipeline for the estimation of the best occlusal relationships between dental arches based on geometric references on 3D models.

First, 3D meshes from IOS were processed and optimized using MeshLab (version 2023.12; ISTI-CNR, Pisa, Italy). Subsequent computational algorithms for alignment and quantitative analysis were developed in MATLAB (version R2023a; MathWorks, Natick, MA, USA), allowing full control over computational procedures and ensuring reproducible results.

The high level of geometric complexity introduced by detailed IOS makes direct application of computational algorithms challenging. To reduce geometrical complexity, the dental surfaces were represented as depth maps preserving the morphological information necessary to assess inter-arch contacts and relationships without reproducing the entire dental geometry, reducing data dimensionality and sensitivity to non-functional variations.

To compute the depth maps, each mesh was sampled on a regular grid of 512 × 512 points and for each point the depth value was calculated using a volumetric ray casting algorithm [37]. At this point, the 3D geometry was projected into a 2D domain while retaining relevant occlusal information, making subsequent processing more efficient [38].

**Figure 1:**
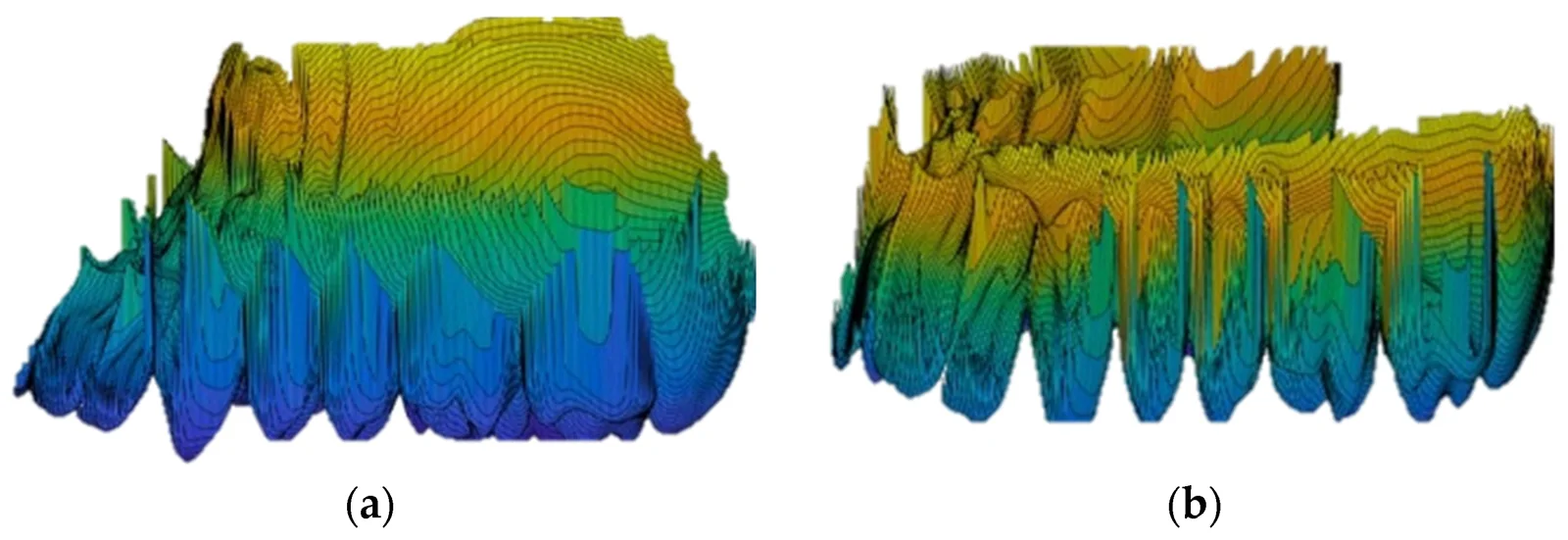
(**a**) Depth map of the maxilla before cropping; left view; (**b**) depth map of the maxilla after cropping; left view.

To isolate relevant geometry from areas of non-interest, the depth maps were cropped (Figure 1).

These simplified models were standardized using principal component analysis (PCA), ensuring consistent orientation and alignment of the datasets in a common reference system [39].

Curvature-based descriptors were then computed on these surfaces, specifically the mean curvature (H) and the Gaussian curvature (K), from which the principal curvatures are derived. These parameters ultimately enabled the calculation of the shape index (S), providing a local classification of surface morphology and allowing the identification of concave, convex, and saddle-shaped regions [40].

**Figure 2:**
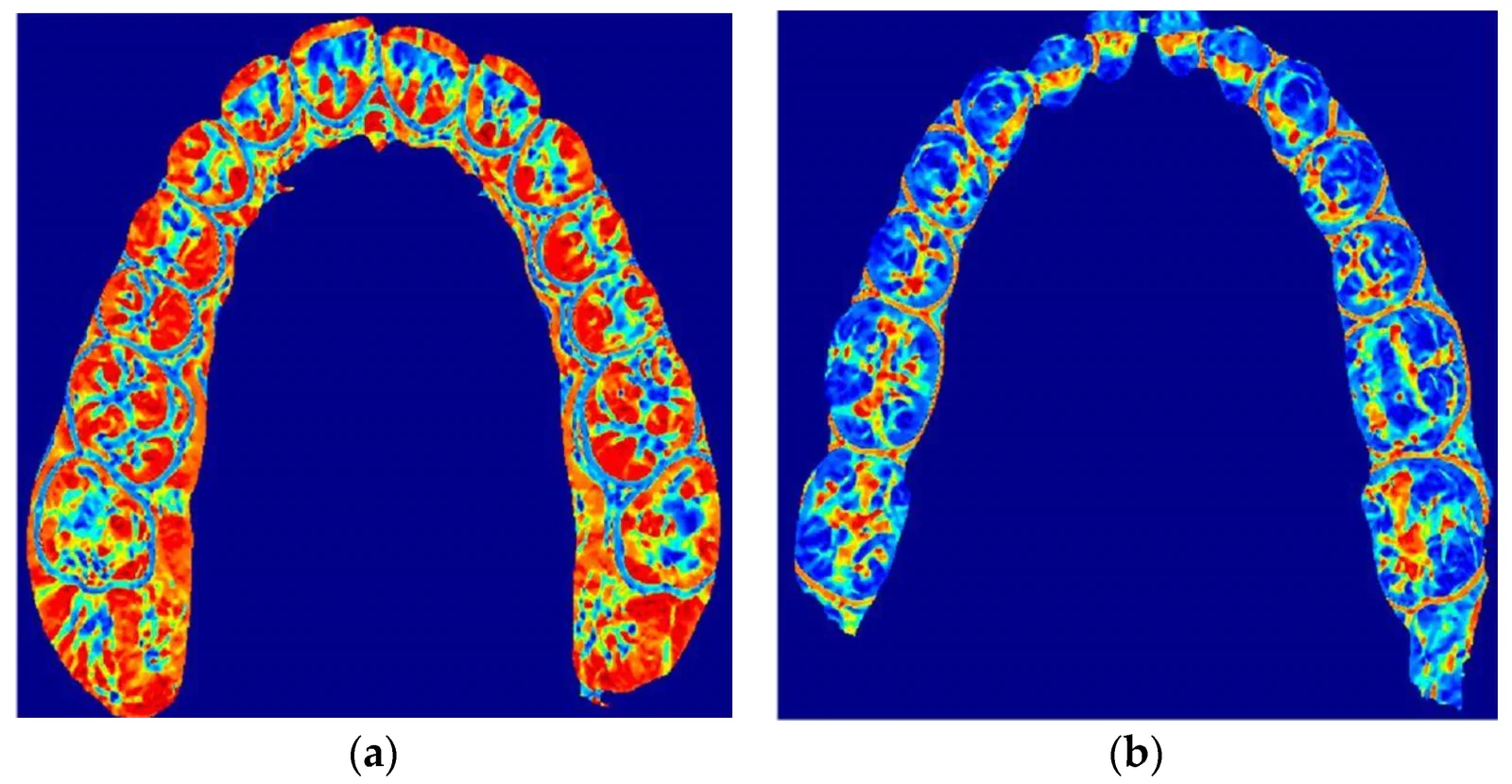
(**a**) Shape index of maxilla, caudal view. Red = convexities; blue = concavities; (**b**) shape index of mandible. Red = concavities; blue = convexities.

To ensure consistency between the upper and lower arches, the sign of the mandibular shape index was inverted to account for the opposite spatial orientation of the mandibular arch (Figure 2). At this point, the shape index values allow the identification of matching points between the two arches, by pairing the concavities of one arch with the convexities of the other, and vice versa.

The resulting maps were stored and used as input features for the subsequent alignment process.

The mandibular arch was considered as a fixed reference, while the maxillary arch was considered as a mobile model. An initial rigid transformation of the arches was obtained using a 2D variant of the Iterative Closest Point (ICP) algorithm, which uses a least-squares approach based on the Kabsch algorithm [41,42]. The algorithm analyses the points of the depth maps, identifies the corresponding points based on the shape index, and subsequently minimizes the distance between them, thereby progressively bringing the two arches closer together by translating them along the x- and y-axis while neglecting translation along the z-axis.

**Figure 3:**
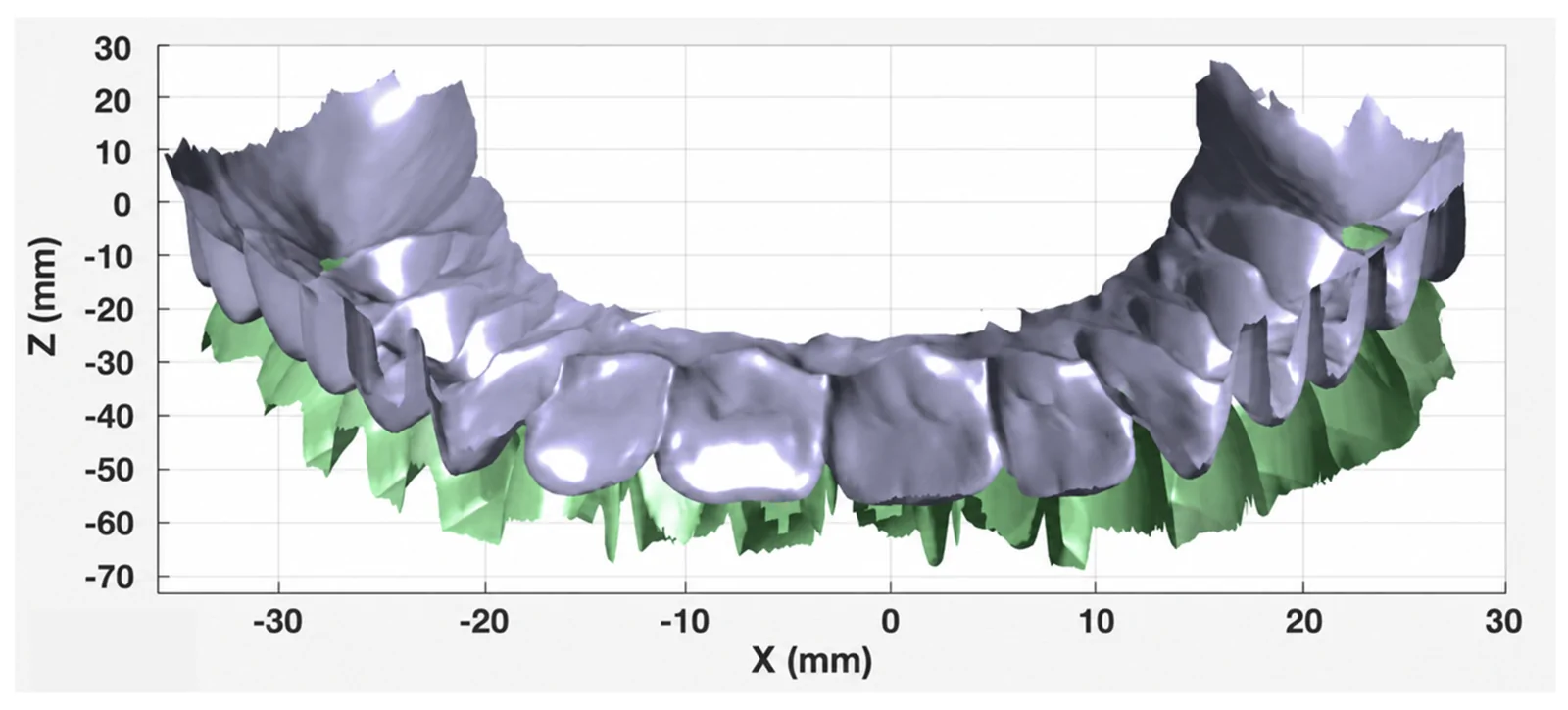
Example of the alignment between corresponding dental arches using 2D ICP on shape index. Frontal view. Grey = maxillary arch’s dental surface; green = mandibular arch’s dental surface.

The adoption of the 2D ICP algorithm, optimally suited for shape information [43], allows the reduction in computational complexity without losing the complex geometrical information of the anatomical models, to which 3D ICP is excessively sensitive. The 2D ICP applied to the shape index enables coupling of the cusps of the maxillary arch with the corresponding fossae of the mandibular arch providing a global reciprocal positioning between maxillary and mandibular models (Figure 3).

To combine the functionality of geometric ICP with the specific functionality of the shape index, a weighted distance between corresponding points on the two arches is defined by combining the Euclidean distance of the ICP with that related to the shape index. This definition is formalized in the following formula:

$$\mathrm{Distance}\;(D)=\left(1-\alpha \right)d_{X}Y+{\alpha d}_{S}$$

where

- $d_{X}Y$ is the Euclidean distance on the XY plane;
- $d_{S}$ is a similarity distance based on the shape index;
- α is the weight accounting for morphological consistency.

The weighting parameter α controls the trade-off between purely geometric ICP alignment (α = 0) and purely morphological matching (α = 1). A heuristic value of α = 0.3 was adopted, assigning a 70% weight to geometric proximity and a 30% weight to morphological correspondence. This value was established internally to prioritize spatial alignment while preserving topological regularization to prevent mesh sliding along smooth dental surfaces. After applying the estimated transformations, a specific refinement phase was implemented to correct the translation on the Z-axis, corresponding to the vertical translation (Z-shift) required to achieve a physiologically consistent inter-arch distance.

**Figure 4:**
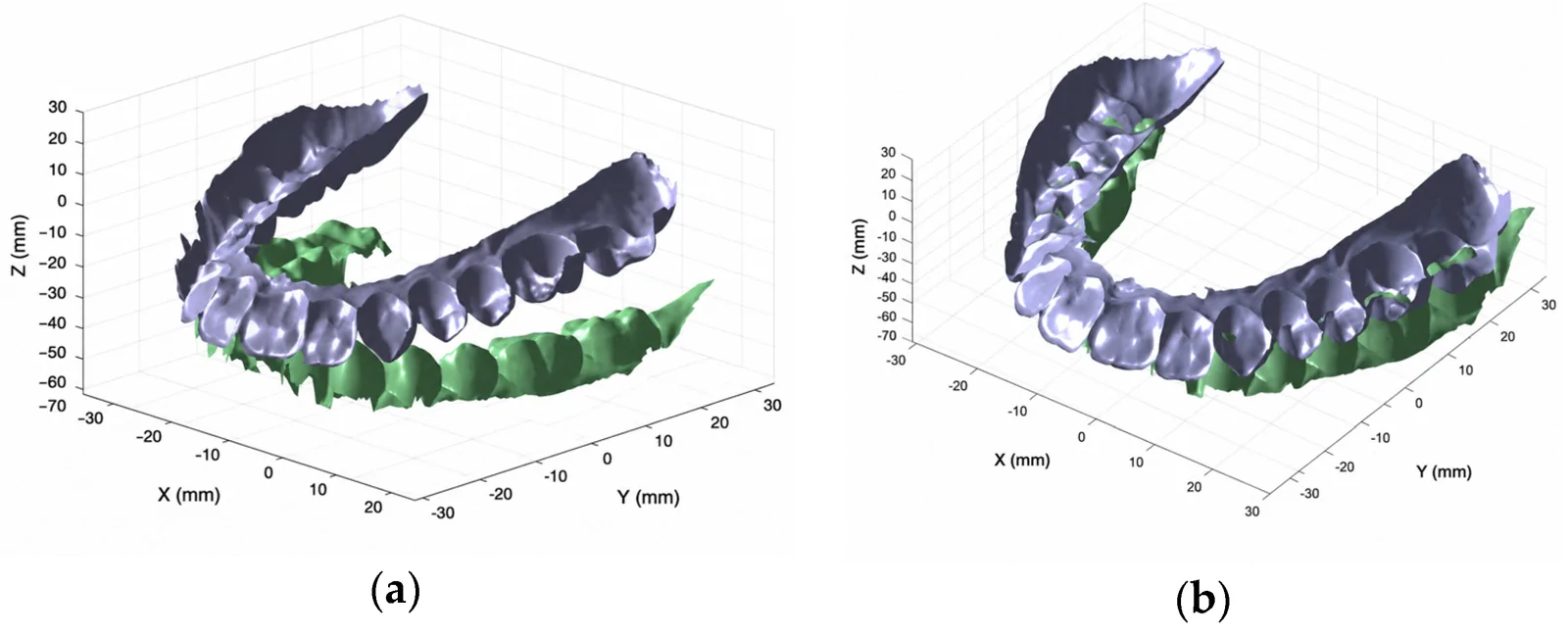
(**a**) Maxillary plane not parallel to the mandibular one; (**b**) maxillary plane parallel to the mandibular one, after correction of the rotation around the X-axis was corrected. Left lateral view. Grey = maxillary arch’s dental surface; green = mandibular arch’s dental surface.

To achieve this, a preliminary correction of the rotation around the X-axis was required in order to obtain a maxillary occlusal plane parallel to the mandibular one, thereby ensuring a uniform Z-axis distance between the corresponding points of the two arches. To do so, the algorithm divides the model along the anteroposterior axis into anterior (incisor) and posterior (molar) regions. Representative surface points on the maxillary arch are selected within these regions, and their vertical distances from the mandibular arch are evaluated. The height difference between the anterior and posterior regions is then used to estimate the corrective inclination, which is subsequently applied as a rotation around the X-axis to compensate for occlusal plane misalignment (Figure 4).

Following tilt correction, the vertical translation (Z-shift) required to achieve a physiologically consistent inter-arch distance is calculated by identifying corresponding points between the two arches via spatial proximity and analyzing their vertical differences. Robust statistical measures such as percentiles are used to reduce the influence of outliers and irregularities on the local surface. The Z-axis translation is applied as the final step before the refinement procedure.

To address cases where excessive inter-arch penetration occurs, a non-penetration constraint was introduced. A penetration threshold was set to 300 μm to account for the physiological compliance of the dentoalveolar complex under occlusal loading. This threshold was selected based on the reported physiological compliance of the tooth–periodontal ligament–alveolar bone complex and was kept constant for all patients [44,45,46]. As with the α parameter, this threshold was established during the software development phase and subsequently kept constant for all patients included in the validation study. However, the framework has been designed to enable the practitioner to modify this value.

**Figure 5:**
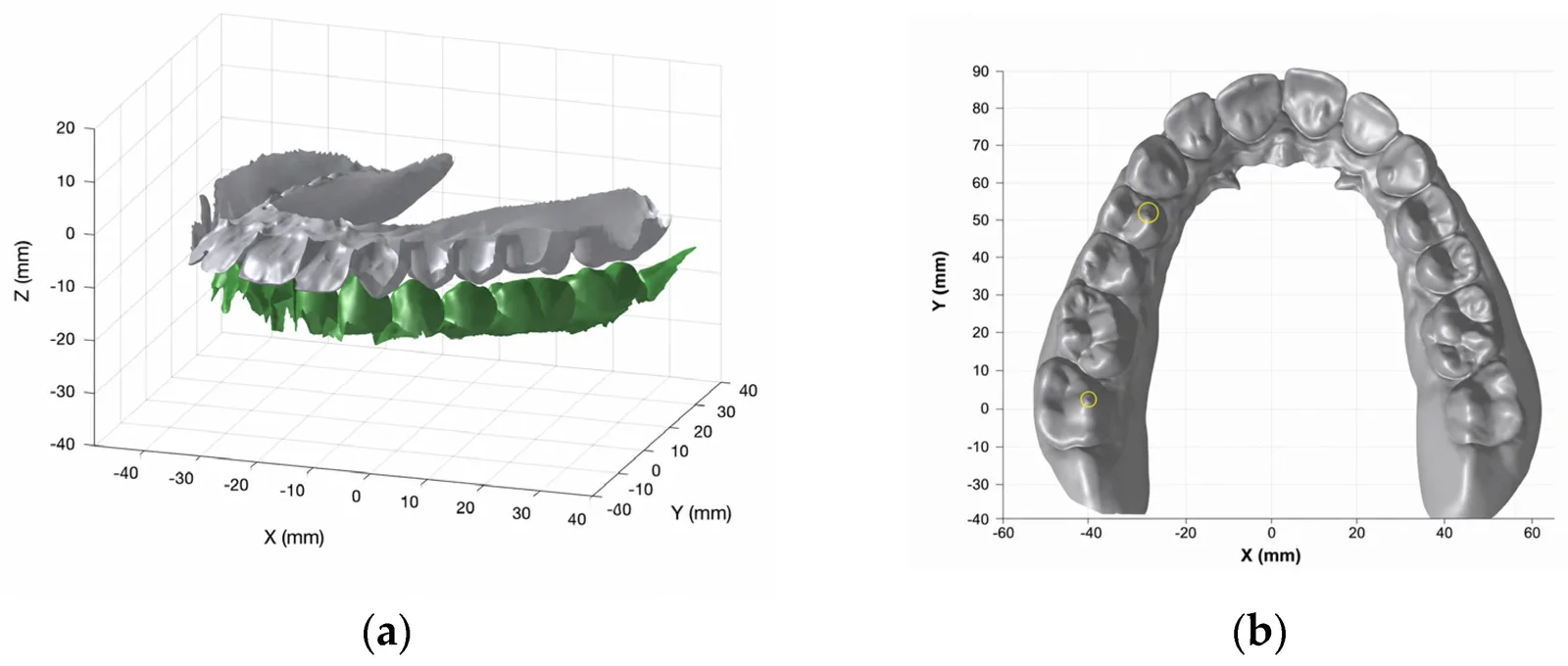
Views of the dental arches after applying the non-penetration constraint. (**a**) Left lateral view; (**b**) caudal view; the points of penetration are highlighted with yellow circles. Grey = maxillary arch’s dental surface; green = mandibular arch’s dental surface.

Two methods for managing the non-penetration constraint are proposed below:

1. The algorithm evaluates the spatial relationship between the maxillary and mandibular arches by measuring the inter-arch distances. Areas of overlap are identified as penetration. Based on this analysis, the extent and distribution of penetrations are calculated to determine the vertical adjustment required to obtain the desired clearance between the arches. The rigid constraint is then applied through a rigid vertical translation of the maxilla model, ensuring a controlled and consistent correction of the inter-arch relationship. The final alignment is subsequently verified to confirm that the required clearance has been achieved. This constraint, as shown in Figure 5, is effective in spacing the arches but provides few stable occlusal contacts.

2. This method, introduced to increase the number of occlusal contacts, formulates the alignment as an iterative optimization problem, in which a rigid transformation of the maxilla is computed to maximize the quality of the occlusion.

The optimization is based on an objective function defined as a weighted combination of three terms:

- A penalty for penetration below a target free space value;
- A contact reward modeled by a Gaussian function centered on the free space value;
- A regularization term to limit non-physiological transformations.

Moreover, a spatial weighting strategy was introduced to modulate the contribution of the three terms along the dental arch, assigning greater importance to the posterior regions, particularly the molars and premolars, while preserving a smaller, yet non-zero, contribution from the anterior region. This weighting is implemented through a logistic function governed by parameters defining the transition point, slope, and relative weighting between the anterior and posterior regions.

At each iteration, the maxillary model is rigidly transformed, and the point vertical spaces are recalculated with respect to the mandibular model. Optimization favors configurations that maintain stable contact while avoiding excessive separation or penetration, resulting in smooth “sliding” behavior between the arches.

**Figure 6:**
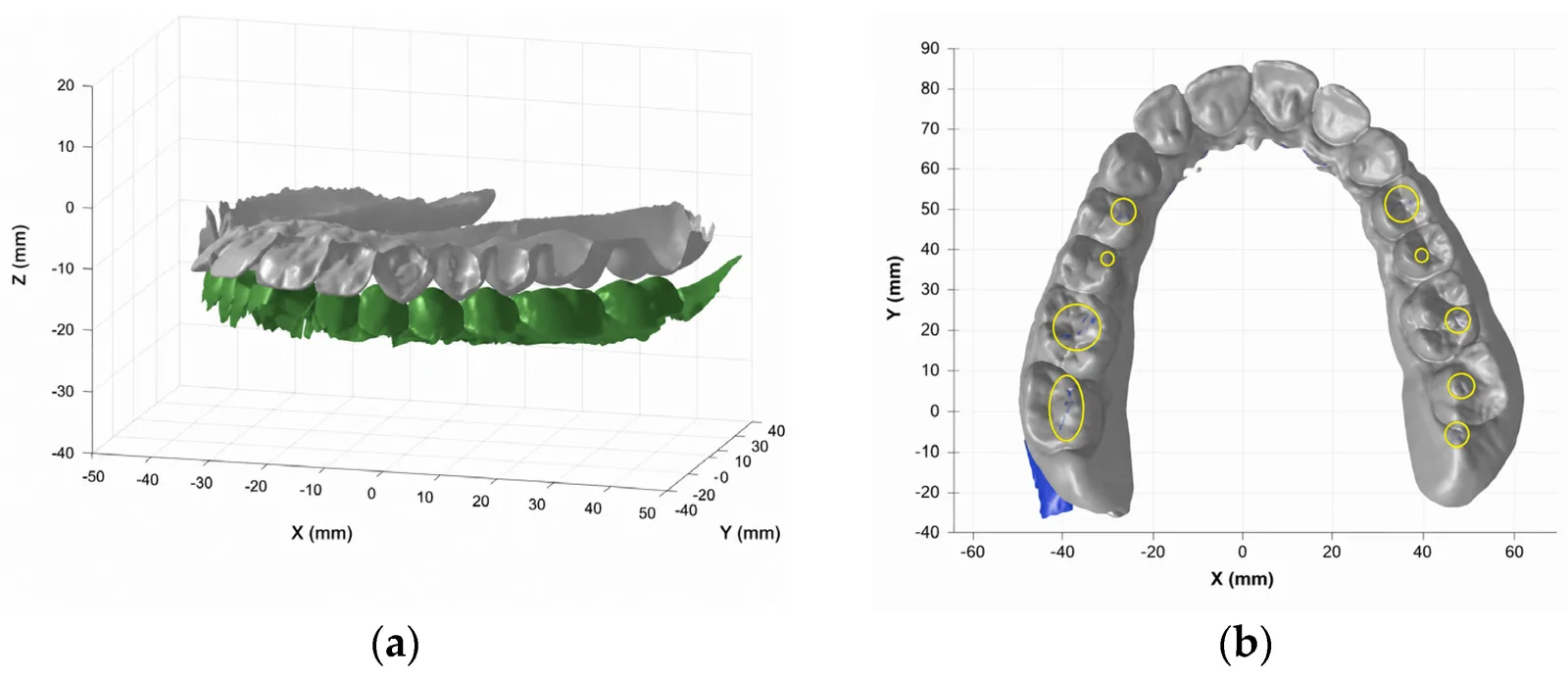
Views of the dental arches after the automatic refinement phase. (**a**) Lateral left view; (**b**) caudal view; the points of penetration are highlighted with yellow circles. Grey = maxillary arch’s dental surface; green = mandibular arch’s dental surface.

The iterative process is performed for up to 900 iterations or until convergence is achieved, typically before the completion of the total number of iterations. This phase follows the initial alignment and allows for a more physiologically realistic occlusion, achieving a controlled compromise between contact stability and non-penetration (Figure 6).

Ultimately, as the latter method was most consistent with the physiological requirements of a stable occlusion, it has been adopted for the subsequent steps of this study. However, the framework has been designed to enable the practitioner to choose between the two approaches.

### 2.4. Manual Stepwise Refinement GUI

A GUI for potential final manual refinement of the occlusion obtained after the automatic phase was implemented.

**Figure 7:**
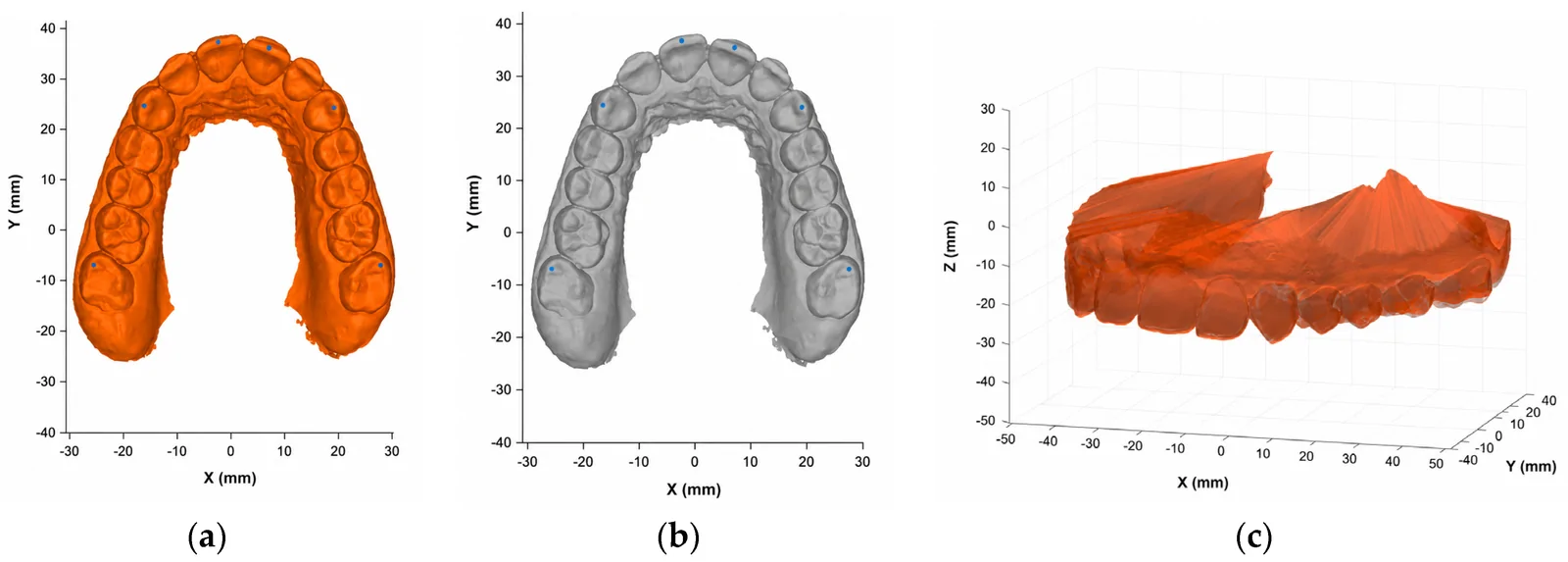
(**a**) Visualization of the operating interface and fiducial points placement on the maxillary arch on the IOS STL model–caudal view; (**b**) visualization of the operating interface and fiducial points placement on the maxillary arch on the depth map generated by the automatic algorithm–caudal view; (**c**) superimposition of the two surfaces to establish point correspondence and registration. Orange = IOS STL maxillary model; Grey = maxillary arch’s dental surface of the depth map. Fiducial points are shown in blue.

To enhance the visualization of the dental arches, the complete models obtained from the IOS were transferred to the current occlusal position defined on the depth maps. A rigid registration procedure based on fiducial points was implemented. This procedure requires the operator to position at least three pairs of corresponding fiducial points to ensure reliable spatial alignment [47] (Figure 7).

Specifically, as shown in Figure 7, the points were selected considering the cusps of the following teeth:

- Mesiobuccal cusps of the right and left second molar;
- Right and left canine cusps;
- The midpoint of right and left maxillary central incisors’ incisal edge.

To improve local accuracy, this initial transformation is refined using traditional ICP together with a multi-resolution strategy that refines the alignment by subsampling the point clouds, and a point-to-plane metric that improves convergence behavior.

Subsequently, the same registration and alignment procedure was applied to the mandibular arch.

**Figure 8:**
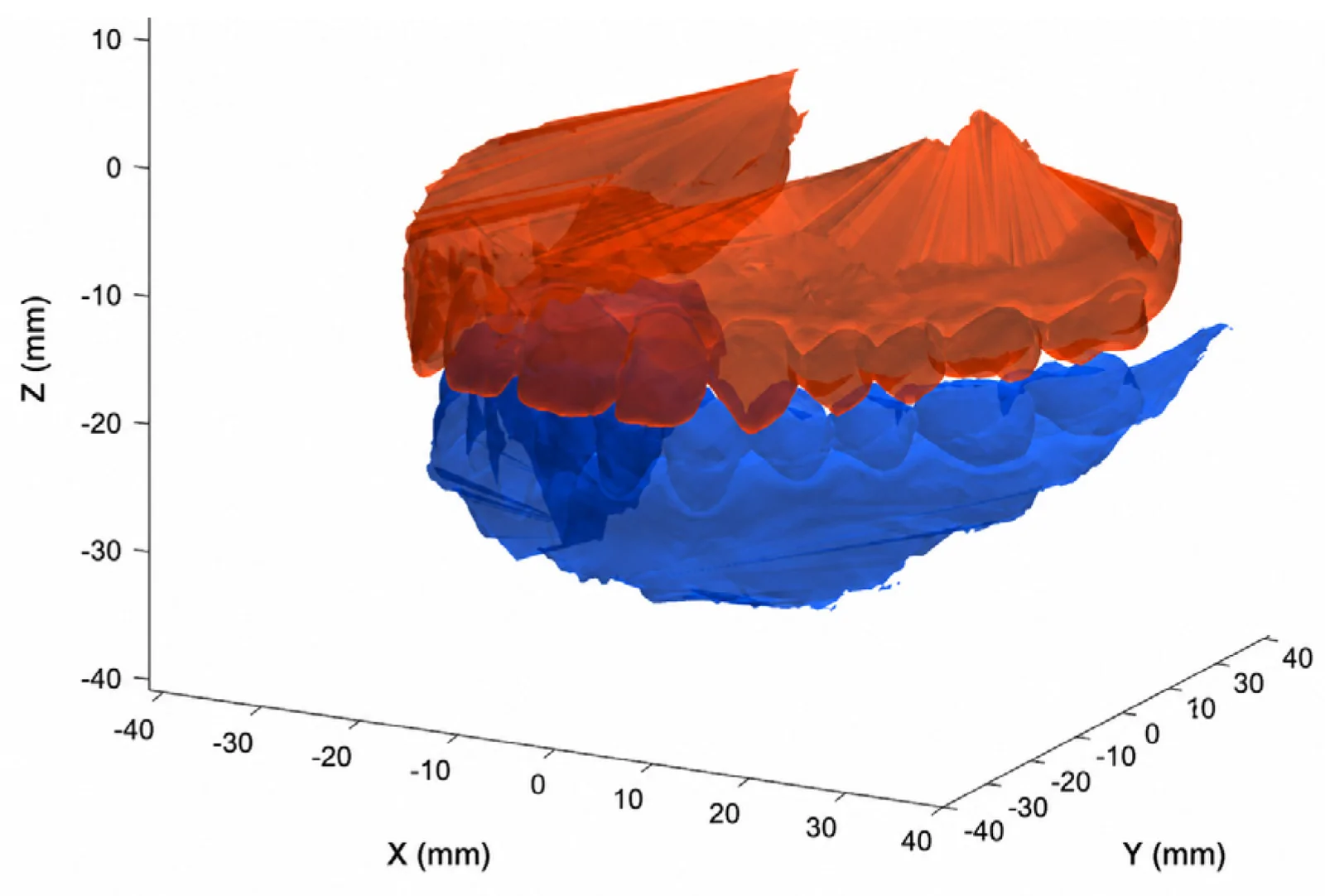
Complete visualization of the arches in occlusion after the automatic digital procedure; left lateral view. Orange = IOS STL maxillary model; blue = IOS STL mandibular model.

At the end of registration procedures, models in final occlusion and with complete geometries to be directly used in preoperative surgical planning were obtained (Figure 8).

**Figure 9:**
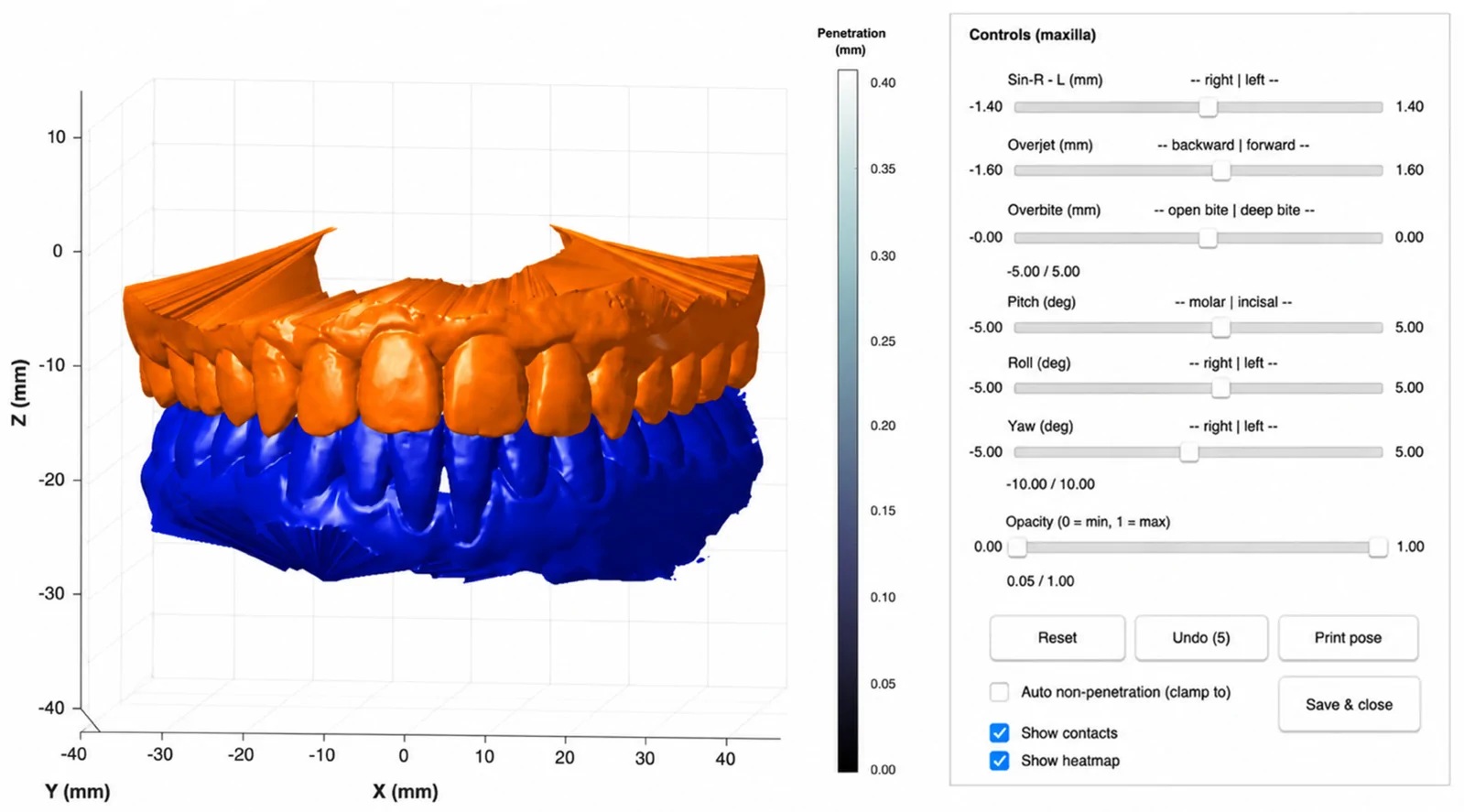
GUI with complete visualization of the arches in occlusion after the automatic digital procedure in frontal view and the manual adjustment interface. Orange = IOS STL maxillary model; blue = IOS STL mandibular model.

At this point, the maxillary and mandibular arches are displayed in an interactive interface with adjustable display parameters. The mandible is held fixed as a reference, while the maxilla is interactively modified through rigid transformations, including translations in mm and rotations in degrees along all axes, controlled via sliders. These controls are expressed using clinically meaningful terminology and indications to ensure intuitive interaction (Figure 9).

The GUI includes tools to restore the configuration, undo the last transformation, view the applied transformation values, and save the result.

Real-time quantitative and qualitative feedback is provided during the interaction. Contacts are visually highlighted, while a heat map dynamically represents the depth of penetration.

The non-penetration constraint can be deactivated in cases where premature contacts prevent the achievement of a stable occlusion. In such situations, the areas of interpenetration are highlighted and identified as regions requiring preoperative occlusal adjustment.

### 2.5. Complete Workflow Validation

To ensure a reproducible validation procedure, controlled misalignments were artificially introduced in the MrO to simulate malocclusion conditions and evaluate the robustness of the full digital workflow with respect to a known reference standard.

The initial misalignment was randomly generated computationally within the MATLAB environment. The magnitude and direction of these transformations were not predefined or standardized across cases, as their sole purpose was to simulate a non-aligned initial condition requiring digital repositioning.

A senior surgeon with high level of expertise in orthognathic surgery from the Maxillofacial Surgery Unit at AOU Città della Salute e della Scienza di Torino executed the digital procedure. After completion of the automatic phase, the surgeon was asked to evaluate whether any further correction was required.

A strict step-by-step protocol was provided, specifying the sequence of evaluations and subsequent occlusal modifications to be performed in an orderly manner [4,10,48]. All adjustments, when needed, were carried out by moving the maxillary arch while maintaining the mandibular arch as the fixed reference.

Initially, the surgeon was instructed to position the models in frontal view and proceed through the revision steps reported in Table 1. Each clinical parameter had to be evaluated and, when considered necessary, corrected before proceeding to the subsequent step.

Then, the surgeon was instructed to rotate the model to the right lateral view (Table 2).

Finally, the surgeon was asked to confirm the result in overall view. The resulting occlusal model was then saved and stored as an .STL file.

In this validation phase, a transformation matrix was calculated to describe the rotational and translational discrepancies between the occlusion obtained using the full digital workflow and the MrO, with the latter being considered the reference standard. Thus, the calculated discrepancies represent the rigid transformation required to align the maxillary position obtained with the digital workflow to that of the MrO, assuming the mandibular models to be perfectly superimposed. For the entire dataset, the matrix was calculated first after the automatic phase alone, and then after the manual stepwise refinement.

It should be noted that, as manual model articulation is inherently operator-dependent and may be subject to inter-operator variability, the manually established occlusion should be regarded as a clinical reference within the context of this study rather than as an absolute, error-free gold standard.

### 2.6. Statistical Analysis

All translational and rotational discrepancies were computed after rigid registration using the same Cartesian coordinate system adopted throughout the analysis. Directional discrepancies (signed mean values) were used to assess systematic bias, whereas mean absolute values were adopted to quantify the magnitude of discrepancies.

Summary statistical indicators were calculated (Microsoft Excel, version 16.109; Microsoft Corp., Redmond, WA, USA) to facilitate the comparison of the obtained results:

- Mean Value (MV), used to characterize the presence and direction of systematic directional bias relative to the manual reference occlusion.

For descriptive purposes, the signed mean difference (bias) and the corresponding 95% limits of agreement were calculated for each translational and rotational discrepancy between VO and MrO, before and after manual refinement. For normally distributed variables, the 95% limits were calculated as the mean difference ± 1.96 standard deviations, whereas for non-normally distributed variables, the empirical 2.5th and 97.5th percentiles were reported. Since the manually established reference occlusion represented the zero-error condition by definition within the context of this study, these statistics were used to describe the distribution of geometric discrepancies and were not interpreted as a formal Bland–Altman assessment of agreement between two independent measurement methods [49].

- Mean Absolute Value (MAV), which represents the average magnitude of the geometric error regardless of their sign.

For each translational and rotational discrepancy, absolute values obtained after the automatic alignment phase were compared with those recorded after the manual refinement phase to determine whether the refinement procedure significantly reduced the error magnitude. Before comparative analysis, the normality of the paired differences was assessed using the Shapiro–Wilk test. Variables with normally distributed paired differences were analyzed using paired Student’s *t*-test, whereas rotation around the Y-axes, which showed a non-normal distribution, was analyzed using the Wilcoxon signed-rank test. Statistical significance was set at *p* < 0.05. Results are reported as mean ± standard deviation (SD), together with paired mean differences, 95% confidence intervals and the corresponding *p*–values.

To provide a better interpretation of the magnitude of the measured discrepancies, predefined acceptance thresholds were considered. Translational deviations within ±2 mm and rotational deviations within ±2° were considered acceptable. Although no universally accepted thresholds currently exist to define the accuracy of a digital occlusion, the ±2 mm/° thresholds have previously been adopted in the literature for the evaluation of occlusal accuracy in orthognathic surgery [50] and were used in this study as reference limits for descriptive interpretation [33,51].

- Translational and rotational Root Mean Square Error (RMSE).

The overall three-dimensional translational and rotational errors were quantified using the 3D root mean square error (RMSE), calculated as:

$$RMSE_{=}\sqrt{\frac{1}{n}\sum_{i=1}^{n}\left(\Delta X_{i}^{2}+\Delta Y_{i}^{2}+\Delta Z_{i}^{2}\right)}$$

where $\Delta X_{i}$, $\Delta Y_{i}$, and $\Delta Z_{i}$ represent the translational/rotational discrepancies along the X-, Y-, and Z-axes for subject i, respectively, and n represents the total number of subjects.

Translational and rotational discrepancies were evaluated within the same Cartesian coordinate system used for the initial model orientation.

For the translational RMSE, we adopted the complete 3D Euclidean spatial norm rather than averaging squared errors across the three axes.

Concerning the rotational RMSE, to address the dependency of Euler-angle decomposition on rotation order, we clarify that the rotation sequence followed the standard XYZ order and axis directions and corresponding movements were as follows:

- X-axis: translational discrepancies along the X-axis represent right–left displacement (positive values indicate rightward displacement). Rotation around the X-axis corresponds to pitch rotation;
- Y-axis: translational discrepancies along the Y-axis represent antero-posterior displacement (positive values indicate posterior displacement). Rotation around the Y-axis corresponds to roll rotation;
- Z-axis: translational discrepancies along the Z-axis represent vertical displacement (positive values indicate superior displacement). Rotation around the Z-axis corresponds to yaw rotation.

Positive rotations around each axis were defined according to the right-hand rule.

## 3. Results

Twenty-four consecutive patients underwent orthognathic surgery with occlusal correction between 3 October 2025 and January 2026 at the Department of Maxillofacial Surgery, AOU Città della Salute e della Scienza di Torino. Three patients were excluded because they underwent segmental maxillary surgery; one of these patients also had fewer than six pairs of opposing teeth in the right hemiarch.

The final study cohort therefore included 21 patients (10 males and 11 females) with a mean age of 26.80 ± 6.60 years. All underwent Le Fort I osteotomy and bilateral sagittal split osteotomy, while 11 (52.4%) also underwent genioplasty. Patient characteristics are summarized in Table 3.

In all cases, following automatic alignment, the surgeon performed at least one manual refinement operation based on the evaluation protocol outlined in Table 1 and Table 2.

**Figure 10:**
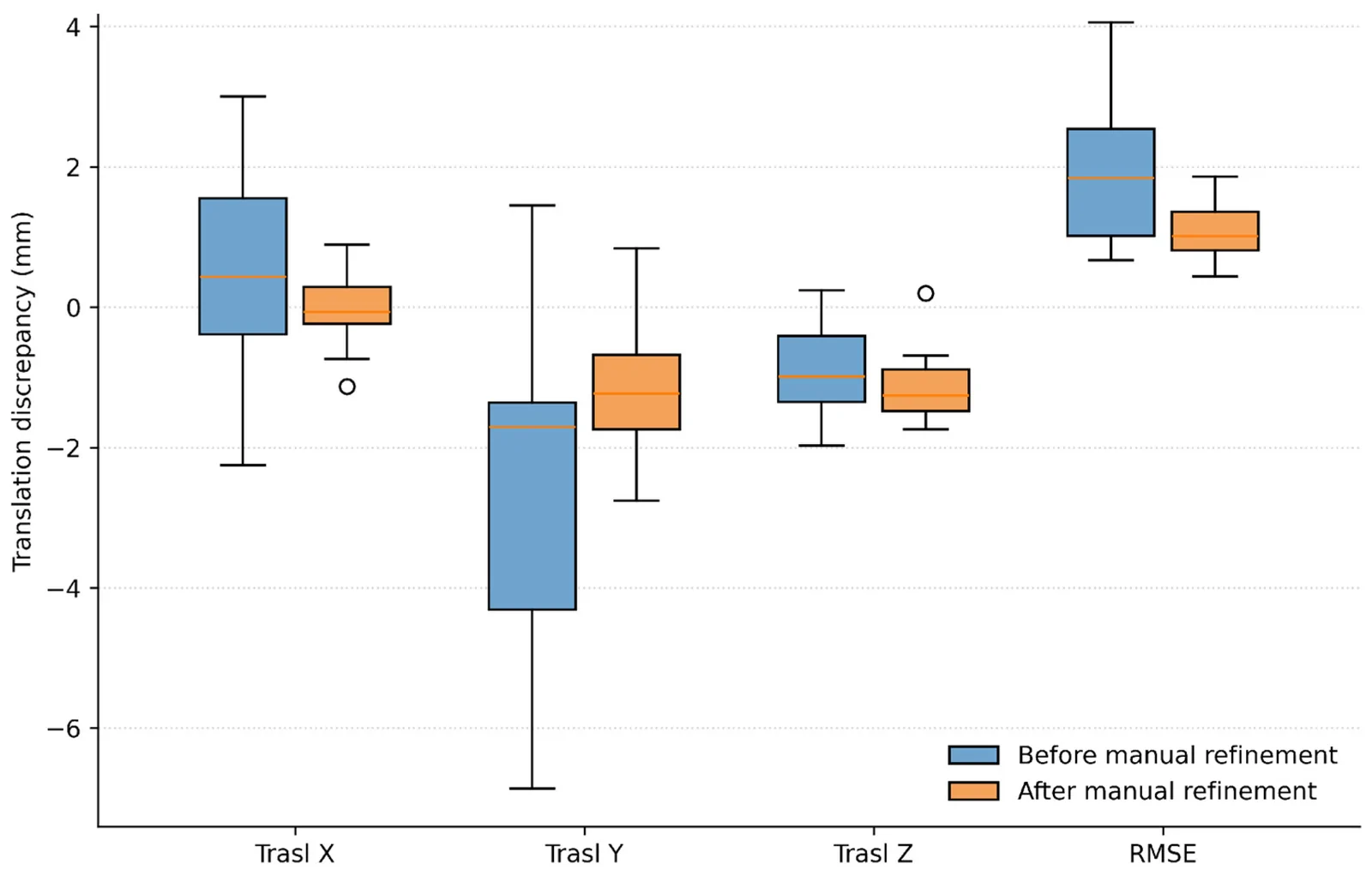
Boxplots showing the distribution of signed translational discrepancies before (blue) and after (orange) manual refinement together, with the root mean square error (RMSE), measured between the occlusion obtained through the digital workflow and the manually refined occlusion (MrO). Boxes represent the interquartile range (IQR), the horizontal line indicates the median, whiskers extend to 1.5 × IQR, and circles denote outliers.

Before manual refinement, the greatest systematic translational bias between VO and MrO was observed along the Y-axis (MV = −2.26 mm) (Table 4, Figure 10). Manual refinement reduced this bias to −1.17 mm. (Table 4, Figure 10). Consistently, the corresponding mean absolute value (MAV), reflecting the magnitude of the discrepancy, decreased significantly from 2.54 mm to 1.26 mm (*p* = 0.002), indicating a smaller systematic anteroposterior deviation (Table 5).

A smaller systematic bias was observed along the Z-axis, with MV values of −0.91 mm and −1.19 mm, before and after refinement, respectively (Table 4, Figure 10). In contrast to the Y-axis, however, after manual refinement the magnitude of the vertical discrepancy increased significantly, with the MAV rising from 0.93 mm to 1.21 mm (*p* = 0.02) (Table 5).

The smallest systematic translational bias was observed along the X-axis, with the MV approaching zero after manual refinement (Table 4, Figure 10). Likewise, the X-axis showed the smallest error magnitude, with the MAV decreasing significantly from 1.12 mm to 0.38 mm following manual refinement (*p* = 0.002) (Table 5).

Overall, the translational RMSE confirmed a significant reduction in the magnitude of translational discrepancies following manual refinement, decreasing from 1.83 mm to 1.09 mm (*p* = 0.001) (Table 5, Figure 10).

**Figure 11:**
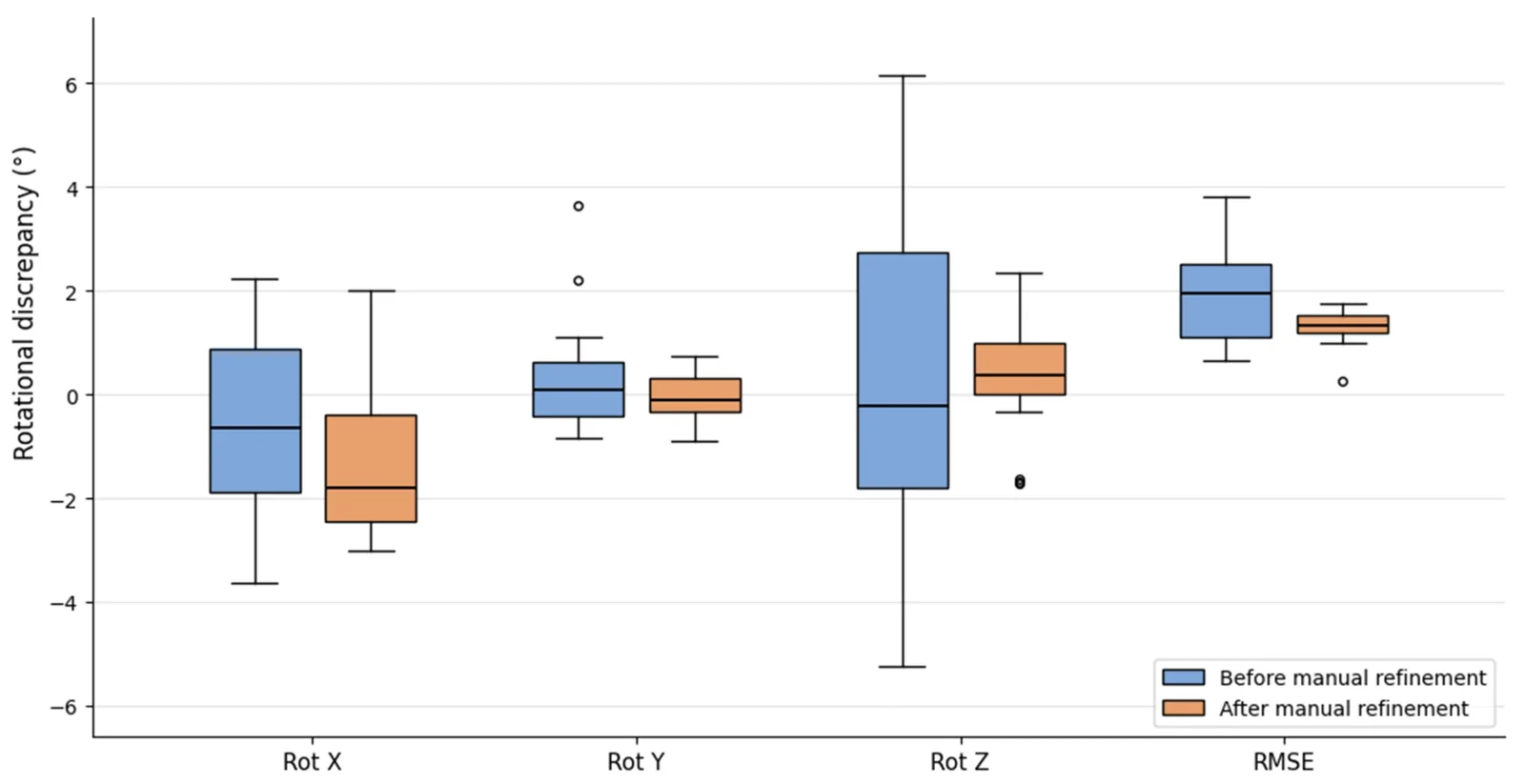
Boxplots showing the distribution of signed rotational discrepancies before (blue) and after manual refinement (orange), together with the root mean square error (RMSE), measured between the occlusion obtained through the digital workflow and the manually refined occlusion (MrO). Boxes represent the interquartile range (IQR), the horizontal line indicates the median, whiskers extend to 1.5 × IQR. Circles denote outliers, whereas filled circles indicate overlapping outliers, corresponding to more than one observation with the same value.

Among the rotational components, the smallest systematic bias was observed around the Y-axis (roll), with an MV of 0.21° and −0.14° before and after refinement, respectively (Table 4, Figure 11). The corresponding MAV decreased from 0.73° to 0.38°; however, this reduction did not reach statistical significance (*p* = 0.13) (Table 5).

The greatest systematic rotational bias was observed around the X-axis (pitch), with MV values of −0.81° before manual refinement and −1.42° after refinement (Table 4, Figure 11). However, the MAV analysis showed no significant improvement in pitch alignment after manual refinement (*p* = 0.09) (Table 5).

The largest rotational error magnitude after automatic alignment was observed around the Z-axis (yaw), despite a relatively small systematic bias (MV = −0.27°). Manual refinement substantially reduced the yaw bias (MV = 0.30°) (Table 4, Figure 11) and, more importantly, significantly decreased the corresponding MAV from 2.61° to 0.83° (*p* = 0.001) (Table 5).

Overall, rotational RMSE confirmed a significant reduction in the magnitude of rotational discrepancies following manual refinement, decreasing from 2.00° to 1.35° (*p* = 0.01) (Table 5).

Extensive patient-level data are reported in Table A1 and Table A2. (Appendix A).

## 4. Discussion

In this study, the separate evaluation of signed mean differences (bias) and mean absolute values (error magnitude) provided additional insight into the behavior of the proposed workflow. Whereas the signed mean difference identified systematic directional tendencies of the virtual occlusion compared to the manual reference occlusion, the MAV quantified the magnitude of the residual discrepancies and their change following manual refinement. Statistical analysis showed that manual refinement significantly reduced the magnitude of all translational and rotational discrepancies between the VO and the MrO, except for rotational errors around the X- and Y-axes (pitch and roll), for which no statistically significant differences were detected.

Following automatic alignment, the negative directional bias observed along the Y-axis indicates a systematic tendency of the algorithm to place the maxillary arch more anteriorly relative to the mandibular arch, thereby overestimating the overjet.

This limitation is consistent with findings reported in the literature. For instance, studies on digital occlusal planning, such as Sabev et al. [50] showed that although digital workflows can achieve clinically acceptable accuracy, discrepancies are more pronounced along the anteroposterior axis. Similarly, Almadi et al. [51] highlighted that the greatest variability in digital occlusion is often associated with sagittal positioning, confirming that this remains a critical challenge for automated systems.

Results obtained after manual refinement revealed a significant reduction in the magnitude of Y-axis translational discrepancies. This observation aligns with previous studies on automatic occlusion reconstruction, such as the algorithm proposed by Chang et al. [50], where it is emphasized that fully automatic approaches struggle to achieve clinically realistic occlusion without operator intervention.

Interestingly, translational discrepancies along the Z-axis significantly increased following manual refinement, contrary to what might be expected. Both before and after manual refinement, the directional bias showed a negative sign, indicating a systematic tendency of the algorithm to position the dental arches closer to each other than the reference occlusion. Following manual refinement, the magnitude of this error significantly increased, suggesting that the correction procedure may have introduced an additional vertical discrepancy.

This increase could be explained by the different constraints governing the two phases of the workflow. During automatic alignment, the non-penetration constraint prevents excessive inter-arch collisions. During manual refinement, the authors suppose that, because the virtual environment does not provide tactile feedback, the clinician cannot directly perceive or control minor interpenetrations between the arches, as would be possible when manipulating physical casts. As a result, the pursuit of improved intercuspation may lead to a slight increase in vertical displacement and local penetrations. This interpretation is supported by clinical studies such as Wong et al. [13] which emphasize that digital models lack physical interaction and therefore cannot inherently reproduce occlusal contact dynamics.

Finally, the smallest translational discrepancies were reported along the X-axis and approached zero following manual refinement. During the refinement phase, the presence of a readily identifiable visual landmark, namely the maxillary and mandibular interincisal lines, enables the operator to visually verify and correct transverse alignment and may account for the consistently low discrepancies observed along the X-axis.

Regarding rotational discrepancies, the largest MAV was recorded for discrepancies around the Z-axis (yaw). Nevertheless, yaw was also the most effectively corrected parameter during manual refinement, with the MAV decreasing from 2.61° to 0.83° (*p* < 0.001).

Although no statistically significant differences were observed for other individual rotational components (pitch and roll), the comparison of the overall rotational RMSE demonstrated a significant reduction in rotational discrepancies, decreasing from 2.00° after automatic alignment to 1.35° following manual refinement (*p* = 0.01). These findings suggest that the refinement process improved the overall rotational accuracy, even though the effect did not reach statistical significance when each rotational axis was analyzed separately.

Furthermore, following manual refinement the dispersion of most discrepancies decreased, with a higher proportion of cases falling within the predefined clinical thresholds.

Overall, the mean absolute values (MAV) of the discrepancies reported after refinement remained below ±2 mm for translations and ±2° for rotations, suggesting that this workflow could achieve clinically acceptable outcomes. Comparable levels of accuracy have also been reported in the literature [51,52].

The proposed semi-automatic workflow represents a compromise between automation and clinical expertise. By combining the efficiency and objectivity of computational alignment with surgeon-guided refinement, it enhances the precision of the final occlusal setup while improving the reproducibility and robustness of the overall planning process. This advantage becomes particularly relevant in complex cases characterized by significant anatomical variability, where purely automatic approaches may struggle to accurately reproduce the desired occlusal relationships.

Several limitations of the present study should be acknowledged.

First, it should be stated that the manually articulated occlusion was the reference workflow against which the automated framework was compared, rather than an absolute clinical gold standard. The present study should be interpreted as a technical agreement analysis between two workflows rather than a clinical validation study. Although translational and rotational discrepancies provide an objective measure of geometrical agreement, they do not directly assess the clinical quality of the resulting occlusion. Future studies with larger cohorts should incorporate clinical endpoints such as occlusal contact analysis, dental relationship parameters, and blinded expert evaluation of surgical planning acceptability.

Second, the sample size was relatively small, and the findings should therefore be considered preliminary. The wide dispersion of discrepancies observed likely reflects the limited cohort size. In addition, the study population included a limited spectrum of anatomical and surgical complexity. Future validation should include more challenging clinical scenarios, such as segmented maxillary arches and atypical segmentation patterns, to better assess the robustness and generalizability of the proposed workflow.

Moreover, neither inter- nor intra-operator reproducibility of the manual refinement phase was evaluated. External validation involving multiple operators and centers will be necessary to assess the generalizability, potential learning effects, usability, and workflow burden of the proposed interface.

Third, a methodological limitation of the current study lies in its internal validation design. The weighting parameter α was determined heuristically within the study sample. Although this value provided a robust balance between geometric proximity and morphological correspondence, it may introduce optimism bias. Moreover, each case was evaluated using a single artificially generated initial misalignment and the penetration threshold was fixed at 0.3 mm throughout the study. Although this threshold was physiologically motivated, its influence on the resulting occlusion was not systematically evaluated. In addition, the uncertainty associated with the registration of the complete STL models to the depth-map-derived position was not assessed; therefore, the contribution of fiducial point placement and ICP registration to the overall measurement error could not be quantified. While these methodological choices were appropriate for this preliminary technical validation of the proposed workflow, future studies should include formal parameter sensitivity analyses, multiple randomized perturbations per patient, and assessments of fiducial registration error, ICP residual error and repeatability to provide a more comprehensive evaluation of the framework’s robustness, stability, and convergence.

Finally, no adjustment for multiple comparisons was applied, as the present study was designed as a preliminary technical validation. Statistical analysis should be regarded as exploratory.

## 5. Conclusions

Overall, this study developed and technically validated a fully digital surgeon-oriented interactive framework for in-house digital occlusal planning in patients undergoing orthognathic surgery. The automatic alignment algorithm provided a reliable initial approximation of the final occlusion. However, the results demonstrated that a manual refinement phase remains necessary to overcome the intrinsic limitations of automatic matching algorithms and to adapt the occlusal setup to patient-specific anatomical and functional requirements.

The findings also highlight the persistent challenges associated with reproducing occlusal relationships within a purely virtual environment, where the absence of tactile feedback may affect the balance between contact optimization and collision control. Consequently, further development of more robust, adaptive, and clinically informed algorithms is warranted to improve performance across the broad spectrum of dentofacial deformities encountered in clinical practice.

Despite these limitations, the proposed workflow represents a meaningful step toward objective, reproducible, and clinically applicable digital occlusal planning. By combining automated computation with surgeon-guided refinement, it offers a practical strategy for integrating digital technologies into routine orthognathic surgical workflows while maintaining clinical oversight of the final occlusal outcome.

## Figures and Tables

**Table 1:** Standardized movement in frontal view.

Clinical Parameter	Aim
1. Interincisal line alignment	Midline discrepancy < 1 mm
2. Maxillary occlusal plane rotation (yaw)	Avoiding crossbite
3. Interincisal line alignment *	Midline discrepancy < 1 mm
4. Occlusal canting (roll)	Achieving vertical symmetry

* Checked again after yaw correction.

**Table 2:** Standardized movement in lateral view.

Clinical Parameter	Aim
1. Canine occlusal relationship	Check for canine class I
2. Overjet	Approximately 2mm
3. Maxillary occlusal plane inclination (pitch)	Achieving at least 3-point teeth contact
4. Overbite	Approximately 2mm
5. Maxillary occlusal plane inclination *	Achieve at least three-point dental contact

* Checked again following overbite correction in cases where the result achieved during phase 3 was modified during phase 4 due to the non-penetration constraint (pitch).

**Table 3:** Clinical dataset: demographic data, pathology and surgical treatment.

N	Age	Gender	Pathology	Surgery
01	24	M	Mn and Mx Asymmetry	LFI, BSSO
02	28	F	Mn Asymmetry + Protrusion, Mx Retrusion	LFI; BSSO; GP
03	19	F	Mn Asymmetry + Protrusion, Mx Retrusion	LFI; BSSO
04	33	F	MX Asymmetry, Mn Asymmetry	LFI; BSSO; GP
05	40	M	Mn Protrusion, Mx Retrusion	LFI; BSSO
06	21	M	Mn Asymmetry + Protrusion, Mx Retrusion	LFI; BSSO; GP
07	24	M	Mn Asymmetry, Mx Retrusion	LFI; BSSO
08	21	F	Mn Protrusion + Asimmetry, Mx Retrusion	LFI; BSSO
09	21	F	Mn Asymmetry + Protrusion, Mx Retrusion	LFI; BSSO
10	27	F	Mn Retrusion, deepbite	LFI; BSSO; GP
11	18	F	Mn Asymmetry, Mx Retrusion	LFI; BSSO
12	26	F	Mn Protrusion, Mx Retrusion	LFI; BSSO
13	40	M	Mn Asymmetry + Retrusion, Mx Retrusion	LFI; BSSO; GP
14	24	M	Mn Retrusion, deepbite	LFI; BSSO; GP
15	27	M	Mn Retrusion + Asimmetry, Mx Retrusion, openbite	LFI; BSSO; GP
16	30	M	Mx Retrusion	LFI; BSSO
17	33	F	Mn Asymmetry + Retrusion, Mx Retrusion	LFI; BSSO; GP
18	20	M	Mn Asymmetry, Mx Retrusion	LFI; BSSO
19	20	F	Mn Retrusion, Mx Retrusion	LFI; BSSO; GP
20	26	F	Mn Retrusion, Mx Retrusion	LFI; BSSO; GP
21	33	F	Mn Asymmetry, Mx Retrusion	LFI; BSSO; GP

M = male; F = female; Mn = mandibular; Mx = maxillary; LFI = le Fort I osteotomy; BSSO = bilateral sagittal split osteotomy; GP = genioplasty.

**Table 4:** Mean signed discrepancies between VO and MrO, corresponding 95% limits of agreement, before and after manual refinement.

Variable	Before Manual Refinement		After Manual Refinement	
	MV ± SD (Bias)	95% Limits	MV ± SD (Bias)	95% Limits
Translation X (mm)	0.32 ± 1.39	−2.40 to 3.04	−0.04 ± 0.49	−1.01 to 0.93
Translation Y (mm)	−2.26 ± 2.1	−6.37 to 1.86	−1.17 ± 0.92	−2.97 to 0.63
Translation Z (mm)	−0.91 ± 0.58	−2.06 to 0.24	−1.19 ± 0.44	−2.06 to −0.32
Rotation X (°)	−0.81 ± 1.71	−4.17 to 2.55	−1.42 ± 1.55	−4.46 to 1.62
Rotation Y (°) *	0.21 ± 1.08	−0.80 to 2.92	−0.14 ± 0.44	−0.84 to 0.590
Rotation Z (°)	−0.27 ± 3.27	−6.68 to 6.15	0.30 ± 1.07	−1.80 to 2.39

* For non-normally distributed variables, the empirical 2.5th and 97.5th percentiles were reported.

**Table 5:** Comparison of mean translational and rotational discrepancies between virtual (VO) and reference occlusion (MrO) before and after manual refinement. Number of patients with discrepancies within the ±2 mm/° threshold and absolute values statistical analysis.

Variable	Before Ref N (%) ≤ 2	After Ref N (%) ≤ 2	Before Ref MAV	After Ref MAV	MD (Post-Pre)	p-Value	95% CI (Post-Pre)
Transl X (mm)	16/21 (76.2)	21/21 (100.0)	1.12	0.38	−0.75	0.002	0.31 to 1.19
Transl Y (mm)	11/21 (52.4)	16/21 (76.2)	2.54	1.26	−1.28	0.002	0.49 to 2.06
Transl Z (mm)	21/21 (100.0)	21/21 (100.0)	0.93	1.21	0.27	0.02	−0.05 to −0.05
Transl RMSE (mm)	14/21 (66.7)	21/21 (100.0)	1.83	1.09	−0.74	0.001	0.36 to 1.14
Rot X (°)	14/21 (66.7)	10/21 (47.6)	1.55	1.96	0.41	0.09	−0.89 to 0.08
Rot Y (°)	19/21 (90.5)	21/21 (100.0)	0.73	0.38	−0.36	0.13 *	−0.04 to 0.76
Rot Z (°)	11/21 (52.4)	20/21 (95.2)	2.61	0.83	−1.77	0.001	−0.84 to 2.70
Rot RMSE (°)	11/21 (52.4)	21/21 (100.0)	2.00	1.35	−0.64	0.01	0.18 to 1.10

Ref = Refinement. N = Number of patients with discrepancies within ±2 mm/°. MAV= Mean Absolute Value. MD = Mean Difference. CI = Confidence Interval. Transl = Translation. Rot = Rotation. RMSE = Root Mean Square Error. * Analyzed using the Wilcoxon signed-rank test due to non-normal distribution.

## Data Availability

The research data supporting the findings of this study are available from the corresponding author upon reasonable request.

## Abbreviations

The following abbreviations are used in this manuscript:

MrO	Manual Reference Occlusion
IOS	Intraoral Scan
3D	Three-Dimensional
STL	Standard Tessellation Language
GUI	Graphical User Interface
ICP	Iterative Closest Point
2D ICP	Two-Dimensional Iterative Closest Point
PCA	Principal Component Analysis
RMSE	Root Mean Square Error
MV	Mean Value
MAV	Mean Absolute Value
SD	Standard Deviation
BSSO	Bilateral Sagittal Split Osteotomy
LFI	Le Fort I Osteotomy
LD	Laterodeviation
GP	Genioplasty
VO	Virtual Occlusion

## Appendix A

**Table A1:** Errors resulting from automatic alignment computational framework.

Subject	Trasl X	Trasl Y	Trasl Z	RMSE_Trasl	Rot X	Rot Y	Rot Z	RMSE_Rot
p01	−0.27	−1.44	−0.05	0.85	−0.64	−0.33	−1.75	1.09
p02	−0.37	−3.04	−0.91	1.84	1.05	−0.01	6.06	3.55
p03	−0.39	−1.41	−0.99	1.02	0.1	−0.85	−4.25	2.5
p04	2.13	0.89	−1.35	1.54	−2.21	−0.15	3.32	2.31
p05	0.49	−4.33	−1.12	2.6	−2.82	−0.52	1.34	1.83
p06	1.01	−2.67	−1.49	1.86	2.23	0.78	6.14	3.8
p07	0.52	−1.56	0.24	0.96	1.81	3.64	3.81	3.22
p08	−1.44	−4.41	−1.3	2.78	−0.66	1.1	−0.08	0.74
p09	−2.11	−4.71	−0.47	2.99	−0.63	0.58	−1.76	1.13
p10	−0.3	−1.36	−1.11	1.03	−2.67	0.23	−0.17	1.55
p11	1.55	−6.86	−0.22	4.06	−0.53	−0.39	−1.94	1.18
p12	1.73	−2.56	−1.31	1.94	−2.66	−0.17	−3.58	2.58
p13	3.0	−1.71	−1.97	2.29	−3.62	0.58	−1.48	2.28
p14	1.55	−1.42	−1.37	1.45	−1.22	−0.68	−0.67	0.89
p15	2.09	−4.38	−1.55	2.94	−0.86	−0.63	3.1	1.89
p16	−2.25	0.05	−0.41	1.32	−3.45	2.2	−3.79	3.22
p17	0.43	0.54	−0.92	0.67	−1.02	0.58	−0.15	0.68
p18	−0.79	−1.22	−0.24	0.85	1.61	0.23	−1.18	1.16
p19	−0.43	1.45	−0.25	0.88	0.81	−0.31	0.77	0.67
p20	−0.12	−4.31	−0.87	2.54	−1.76	−0.74	−5.23	3.21
p21	0.64	−2.98	−1.45	1.95	0.1	−0.71	−4.14	2.43
MV ± SD	0.32 ± 1.39	−2.26 ± 2.10	−0.91 ± 0.58		−0.81 ± 1.71	0.21 ± 1.08	−0.27 ± 3.27	
MAV ± SD	1.12 ± 0.84	2.54 ± 1.73	0.93 ± 0.55	1.83 ± 0.91	1.55 ± 1.06	0.73 ± 0.81	2.61 ± 1.91	2.00 ± 1.01

RMSE = Root Mean Square Error; MV = Mean Value; MAV = Mean Absolute Value; SD = Standard Deviation.

**Table A2:** Errors resulting after standardized manual refinement phase.

Subject	Trasl X	Trasl Y	Trasl Z	RMSE_Trasl	Rot X	Rot Y	Rot Z	RMSE_Rot
p01	0.89	−1.23	−0.74	0.98	−1.85	−0.09	2.35	1.72
p02	−1.13	−0.68	−0.82	0.89	1.84	−0.09	1.06	1.23
p03	−0.74	−0.9	−1.2	0.96	−2.76	−0.06	0.49	1.62
p04	0.4	−0.57	−1.22	0.81	−2.63	−0.33	0.1	1.53
p05	−0.14	−1.07	−1.37	1.01	−2.23	−0.74	1.94	1.76
p06	−0.02	−2.61	−1.39	1.71	1.79	−0.78	1.33	1.36
p07	0.54	−2.76	−1.56	1.86	−2.18	0.19	−0.34	1.28
p08	0.08	−2.07	−1.42	1.45	−2.06	0.29	0.08	1.2
p09	−0.44	−1.45	−1.61	1.28	−1.78	0.74	0.37	1.13
p10	−0.07	−2.31	−1.13	1.49	−3.01	0.43	0.03	1.76
p11	0.47	−0.73	−1.26	0.88	−0.33	−0.18	−1.67	0.99
p12	0.17	−1.45	−1.74	1.31	−2.58	0.44	0.38	1.53
p13	0.29	−2.07	−1.31	1.42	−2.53	0.01	−0.19	1.46
p14	−0.16	−0.71	−1.13	0.78	−1.82	−0.89	0.53	1.21
p15	−0.24	−1.58	−1.48	1.26	−2.72	−0.13	−0.1	1.57
p16	−0.36	−0.14	−0.89	0.56	−1.47	−0.35	0.98	1.04
p17	−0.07	−0.24	−0.89	0.54	−0.42	0.38	−1.62	0.99
p18	−0.22	0.84	−0.69	0.64	−1.33	−0.65	1.22	1.11
p19	−0.73	0.13	0.2	0.44	2.01	−0.32	0.76	1.25
p20	−0.03	−1.74	−1.58	1.36	−2.41	−0.19	0.22	1.4
p21	0.7	−1.24	−1.67	1.27	−1.31	−0.61	−1.72	1.3
MV ± SD)	−0.04 ± 0.49	−1.17 ± 0.92	−1.19 ± 0.44		−1.42 ± 1.55	−0.14 ± 0.44	0.30 ± 1.07	
MAV ± SD	0.38 ± 0.31	1.26 ± 0.78	1.21 ± 0.39	1.09 ± 0.39	1.95 ± 0.71	0.38 ± 0.27	0.83 ± 0.71	1.35 ± 0.25

RMSE = Root Mean Square Error; MV = Mean Value; MAV = Mean Absolute Value; SD = Standard Deviation.
